# New Cellular Interactions Due to the Radioprotective Effect of N-Acetylcysteine in a Model of Radiation-Induced Pancreatitis

**DOI:** 10.3390/ijms26115238

**Published:** 2025-05-29

**Authors:** Grigory Demyashkin, Matvey Vadyukhin, Vladimir Shchekin, Tatyana Borovaya, Olga Zavialova, Dmitriy Belokopytov, Kirill Silakov, Petr Shegay, Andrei Kaprin

**Affiliations:** 1 Department of Digital Oncomorphology, National Medical Research Centre of Radiology, 2nd Botkinsky Pass., 3, Moscow 125284, Russia; vma20@mail.ru (M.V.); dr.shchekin@mail.ru (V.S.); tbor27@yandex.ru (T.B.); beldimbur@gmail.com (D.B.); dr.shegai@mail.ru (P.S.); kaprin@mail.ru (A.K.); 2 Laboratory of Histology and Immunohistochemistry, Institute of Translational Medicine and Biotechnology, I.M. Sechenov First Moscow State Medical University (Sechenov University), Trubetskaya St., 8/2, Moscow 119048, Russia; maxwf1337@mail.ru; 3 Research and Educational Resource Center for Immunophenotyping, Digital Spatial Profiling and Ultrastructural Analysis Innovative Technologies, Peoples’ Friendship University of Russia (RUDN University), Miklukho-Maklaya Str. 6, Moscow 117198, Russia; path.silakov@gmail.com; 4 Department of Urology and Operative Nephrology, Peoples’ Friendship University of Russia (RUDN University), Miklukho-Maklaya Str. 6, Moscow 117198, Russia

**Keywords:** electron irradiation, pancreas, insulin resistance, pancreatic islets, inflammation

## Abstract

Ionizing radiation at early stages leads to radiation-induced death of Langerhans islet cells and acinar cells, resulting in the development of acute/subacute pancreatitis. Conducting studies on radiation-induced changes in the pancreas following electron beam irradiation appears to be of great interest, and the evaluation of radioprotective agents for safeguarding normal tissues from radiation is equally important. The aim of this study was to preclinically investigate the antioxidant properties of N-Acetylcysteine in an animal model of radiation-induced pancreatitis over a three-month period. In this study, it was proven for the first time that even electrons can lead to characteristic signs of radiation-induced pancreatitis, the degree of which was assessed based on the levels of insulin, glucose, and amylase. Thus, conducting electron therapy also increases the risks of insulin resistance, as well as X-ray and gamma radiation. For the first time, a comprehensive analysis of biochemical, morphological, and immunohistochemical markers in the pancreas of a large cohort of electron-irradiated animals was conducted, including both acute and delayed effects of electron exposure. The crucial role of interleukins in shaping both the cellular and vascular components of the inflammatory response was identified. Additionally, the radioprotective properties of N-Acetylcysteine during electron irradiation of the pancreas were evaluated for the first time, and its effectiveness in reducing both acute and late complications of electron therapy was demonstrated. Thus, it can be concluded that N-Acetylcysteine is capable of effectively suppressing the inflammatory response in the pancreas.

## 1. Introduction

The impact of ionizing radiation in the treatment of malignant neoplasms of the abdominal organs sometimes leads to damage to normal pancreatic cells and the development of radiation-induced pancreatitis [1]. In the long term, this condition is associated with the development of exocrine and endocrine insufficiency and is accompanied by chronic inflammation, parenchymal fibrosis, and diabetes mellitus [2]. Several retrospective cohort studies have been conducted to prove this fact [1,2,3]. Despite the small sample of pediatric and adult patients, a reliable increase in the risk of developing pancreatitis in the early stages and diabetes mellitus as a complication was found, reaching 16% or more. Particularly serious changes were observed during radiotherapy of the pancreatic tail at doses above 10 Gy [1].

In the modern scientific literature, there is evidence of diabetes mellitus developing as a late complication of radiation therapy for pancreatic cancer in children under the age of two. As they grow older, an increased incidence of insulin resistance and type II diabetes mellitus has been observed, reaching approximately 7% of cases by the age of 45 and older [3]. At the same time, some authors do not exclude the role of radiation-induced damage to subcutaneous adipose tissue after abdominal irradiation in the process of developing relative β-cell insufficiency and insulin resistance in the long term [4].

Exposure to X-ray and γ radiation leads to the development of radiation-induced pancreatitis signs, such as hemorrhages, necrosis, edema, and inflammatory infiltration of varying severity [5]. On the contrary, electrons have a more sparing effect on normal cells compared to other types of ionizing radiation (X-rays and γ-waves), while maintaining a high level of effectiveness against atypical cells [6].

Most authors agree that ionizing radiation at early stages leads to radiation-induced death of Langerhans islet cells and acinar cells, resulting in the development of acute/subacute pancreatitis with focal necrosis, cellular inflammatory infiltration, hemorrhages, edema, and signs of parenchymal fibrosis, among other effects. This condition is associated with the activation of direct and indirect mechanisms of radiation damage. The direct mechanism involves the formation of chromosomal aberrations due to the direct impact of electrons on the strands of nuclear and circular mitochondrial DNA, leading to the formation of single- and double-strand breaks and cross-links. This process activates cascades of the intrinsic and ceramide pathways of apoptosis, as well as mitochondrial permeability transition-driven necrosis (MPT-driven necrosis) [7,8].

Additionally, the impact of electrons leads to the radiolysis of extra- and intracellular water, resulting in the formation of free radicals, including reactive oxygen species (ROS), reactive nitrogen species (RNS), and lipid peroxidation products. This process causes damage to intracellular macromolecules (proteins, lipids, etc.) and disrupts the permeability of the cytoplasmic membrane. These effects are associated with the development of oxidative stress, impairment of the endogenous redox system, depletion and decreased activity of antioxidant defense mechanisms, and toxic cell damage, ultimately leading to the activation of the extrinsic apoptosis cascade. Thus, in response to radiation exposure, three apoptosis pathways are activated: the intrinsic pathway (via p53 and caspase-9), the extrinsic pathway (via tumor necrosis factor-α and caspase-8), and the ceramide pathway (via sphingomyelin hydrolysis involving acid sphingomyelinase) [7].

Most authors agree that inflammation is triggered in response to cell death through both direct [8] and indirect pathways of radiation damage, leading to the recruitment of immune cells (primarily macrophages and T-lymphocytes) to the irradiated area. In response to the presence of damage-associated molecular patterns (DAMPs), these immune cells synthesize large amounts of pro-inflammatory cytokines, including IL-1, IL-2, IL-6, IL-8, and IL-33, as well as inflammatory factors, such as TNF-α, IFN-γ, NF-κB, and SMAD2/3. Additionally, they produce growth factors, such as TGF-β and PDGF, among others. As a result, immune cells, including CD3+ T-lymphocytes, CD68+ macrophages, mast cells, and others, migrate to the pancreas [9].

An interesting area is the study of radioprotective agents capable of preventing oxidative stress and toxic cell damage caused by free radicals, such as melatonin, curcumin, etc. [10]. Some researchers have demonstrated the radioprotective activity of Amifostine, which is the only recommended drug for clinical use to protect normal tissues from radiation exposure [11]. However, this drug has several disadvantages, including an inconvenient route of administration, high cost, and toxicity, among others. Therefore, the development of preventive strategies for radiation-induced complications remains a relevant and important topic.

One of the pharmaceutical agents that may possess antioxidant properties is N-Acetylcysteine (N-AC) as an off-label application. Some studies have demonstrated its role in reducing levels of caspase-3, malondialdehyde (a marker of lipid peroxidation), and the expression of inflammatory factors (TNF-α, NF-κB). Additionally, N-AC has been shown to stimulate endogenous antioxidant defense by increasing glutathione levels [12]. Moreover, some authors have identified positive effects of N-AC in oxidative stress models of KrasG12D-induced pancreatitis and hepatitis, where this compound reduced the expression levels of TNF-α and NF-κB. This led to reduced inflammation and decreased cell death [13].

Thus, conducting studies on radiation-induced changes in the pancreas following electron beam irradiation, a promising and less-explored method of radiation therapy, appears to be of great interest. Additionally, the development and evaluation of radioprotective agents for safeguarding normal tissues from radiation exposure are equally important. One such approach involves the administration of antioxidant agents, such as N-Acetylcysteine, to mitigate radiation-induced damage.

## 2. Research Objective

The aim of this study is to preclinically investigate the antioxidant properties of N-Acetylcysteine in an animal model of radiation-induced pancreatitis in the context of its antioxidant properties, effects on downstream molecular pathways, pancreatic structure and function, cell cycle, and inflammation, and to evaluate these changes over a three-month period.

## 3. Results

### 3.1. Biochemical Assay

**Figure 1:**
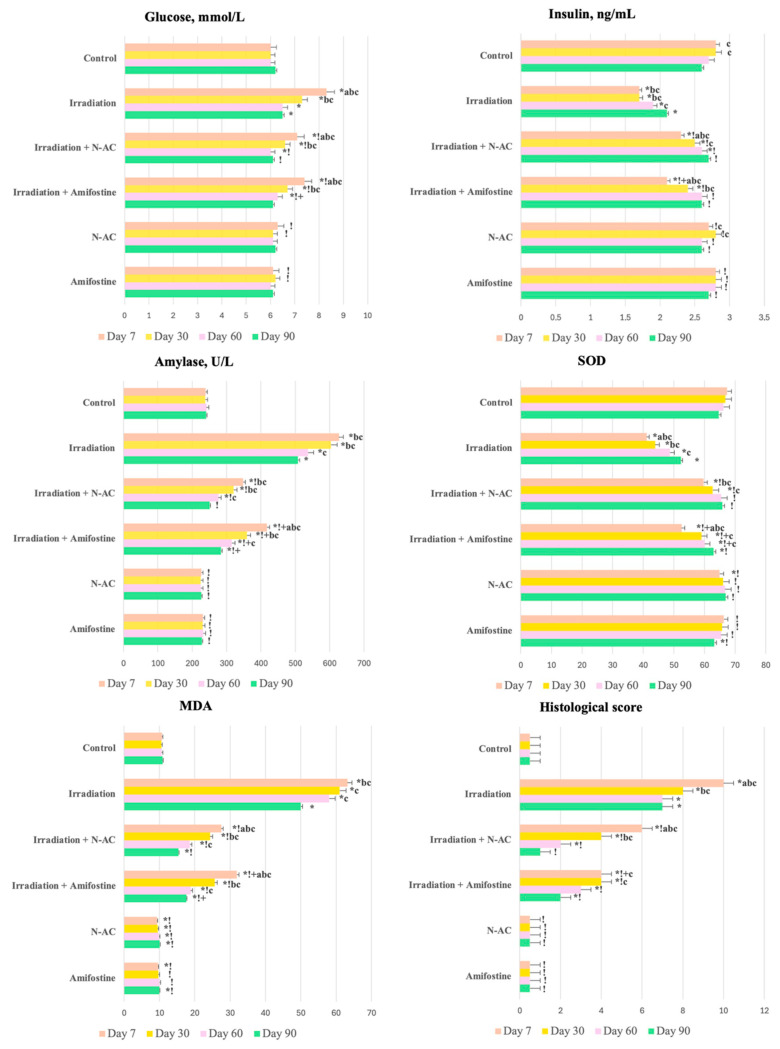
Results of biochemical blood assays (glucose, insulin, amylase), superoxide dismutase and malondialdehyde levels, and the severity of pancreatitis (total score based on histological criteria) in the control and experimental groups on days 7, 30, 60, and 90. Data are presented as mean values (ranges), with Kruskal–Wallis test and Mann–Whitney U-test values provided. SOD—superoxide dismutase, MDA—malondialdehyde, N-AC—N-Acetylcysteine. Statistically significant differences (*p* < 0.05): a—day 7 vs. day 30 within the same group, b—compared to day 60 within the same group, c—compared to day 90 within the same group; *—compared to control; !—compared to the irradiation group at the same time point; +—Amifostine vs. N-AC.

In the study of glucose, insulin, and amylase levels in the blood of group II, an increase in the glucose and amylase levels by 38.3% and 2.6 times, respectively, was observed compared to the control, while the insulin levels in this group were reduced by 40%. At the same time, the administration of N-AC before irradiation led to less pronounced changes in the studied biochemical parameters: the glucose and amylase levels increased by 18.3% and 45.5%, respectively, while the insulin levels decreased by 18.0% (Figure 1). Interestingly, N-AC was more effective than Amifostine in the early stages, whereas in the irradiation group, no positive trends in the studied parameters were observed throughout the experiment.

### 3.2. Oxidative Stress Markers

In the pancreatic tissue homogenate after fractional electron irradiation, an increase in the MDA levels by 5.9 times and a decrease in the SOD levels by 39.1% were observed. Less pronounced changes in the MDA and SOD levels were found in group III, with MDA increasing 2.5 times and SOD decreasing by 21.5% compared to the control. In group V, the MDA level was significantly lower (by 13.1%) than in the control (Figure 1). In the early stages, N-AC was more effective than Amifostine, whereas in the irradiation group, no positive trends in the studied parameters were observed throughout the experiment.

### 3.3. Morphometric Analysis

**Figure 2:**
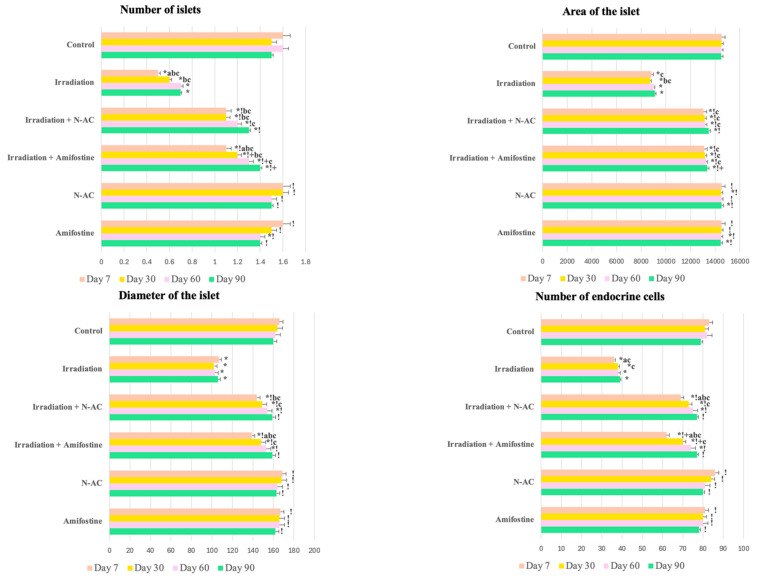
Morphometric analysis of pancreatic fragments in the control and experimental groups on days 7, 30, 60, and 90: number of islets (per 1 mm^2^), islet area (µm^2^), islet diameter (µm), and percentage of endocrine cells in the islet. Data are presented as mean values (ranges), with Kruskal–Wallis test and Mann–Whitney U-test values provided. SOD—superoxide dismutase, MDA—malondialdehyde, N-AC—N-Acetylcysteine. Statistically significant differences (*p* < 0.05): a—day 7 vs. day 30 within the same group, b—compared to day 60 within the same group, c—compared to day 90 within the same group; *—compared to control; !—compared to the irradiation group at the same time point; +—Amifostine vs. N-AC.

In the morphometric study of pancreatic structures, group II showed a significant reduction in key morphometric parameters compared to the control: a decrease in the number (by 69.7%), area (by 38.3%), and diameter (by 35.5%) of pancreatic islets, as well as a reduction in the total number of endocrine cells in a single islet by 47.0%. In group III, the pre-administration of N-AC led to a decrease in the number (by 31.3%), area (by 8.6%), and diameter (by 13.3%) of pancreatic islets, as well as a reduction in the total number of endocrine cells in a single islet by 14.0% compared to the control (Figure 2). In the early stages, N-Acetylcysteine was more effective than Amifostine in some parameters, whereas in the irradiation group, no positive trends in the studied parameters were observed throughout the experiment.

### 3.4. Histological Patterns

**Figure 3:**
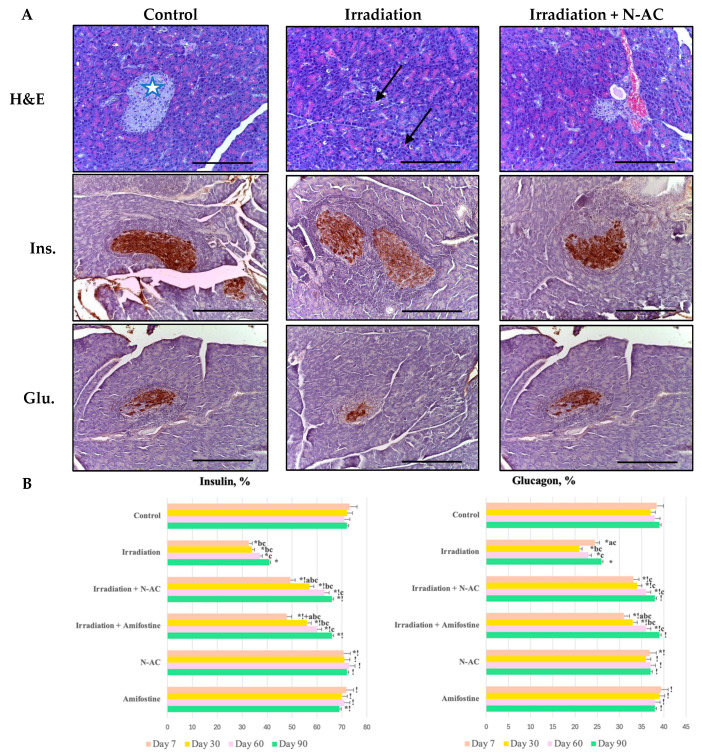
Pancreas of the control and experimental groups (**A**). Top row—Morphological picture, hematoxylin and eosin (H&E) staining, magn. ×200. Cellular inflammatory infiltration (arrow), pancreatic islet (asterisk). Middle row—Immunohistochemical picture with antibodies to insulin (Ins), magn. ×200. Bottom row—Immunohistochemical picture with antibodies to glucagon (Glu), magn. ×200. Scale bar—50 μm. Distribution of the number of positive cells in immunohistochemical reactions with antibodies against insulin and glucagon on days 7, 30, 60, and 90 (**B**). Data are presented as mean values (ranges), with Kruskal–Wallis test and Mann–Whitney U-test values provided. SOD—superoxide dismutase, MDA—malondialdehyde, N-AC—N-Acetylcysteine. Statistically significant differences (*p* < 0.05): a—day 7 vs. day 30 within the same group, b—compared to day 60 within the same group, c—compared to day 90 within the same group; *—compared to control; !—compared to the irradiation group at the same time point; +—Amifostine vs. N-AC.

When studying the morphology of the pancreas one week after local electron irradiation, group II exhibited disrupted histoarchitecture characterized by signs of subacute radiation-induced pancreatitis: cystic expansion of most acini, vacuolization, focal necrosis, hemorrhages, edema, and lymphocytic infiltration. Because of N-AC administration, a slight reduction in the severity and extent of pancreatic damage (by 1–2 points) was observed in group III (Figure 3). In the first week, N-AC was more effective than Amifostine as a radioprotector. However, the degree of histological changes did not differ significantly at later stages between the groups receiving radioprotectors. In the irradiation group, no positive trends in the studied parameters were observed throughout the experiment.

### 3.5. Immunohistochemical Assay

The endocrine cells in the pancreatic islets of the control group exhibited staining with antibodies against insulin (3 points) and glucagon (2 points) based on the results of the IHC study. One week after fractional electron irradiation, a decrease in insulin expression (by 2.2 times; 2 points) and glucagon expression (by 1.5 times; 1 point) was observed compared to the control. In the group that received N-AC before irradiation, the number of immunopositive endocrine cells was close to the control values one week later: insulin was 1.5 times lower (2–3 points) and glucagon was 1.2 times lower (2 points) (Figure 3). In the early stages, N-AC was more effective than Amifostine in protecting β-cells (insulin), whereas in the irradiation group, no positive trends in the studied parameters were observed throughout the experiment.

**Figure 4:**
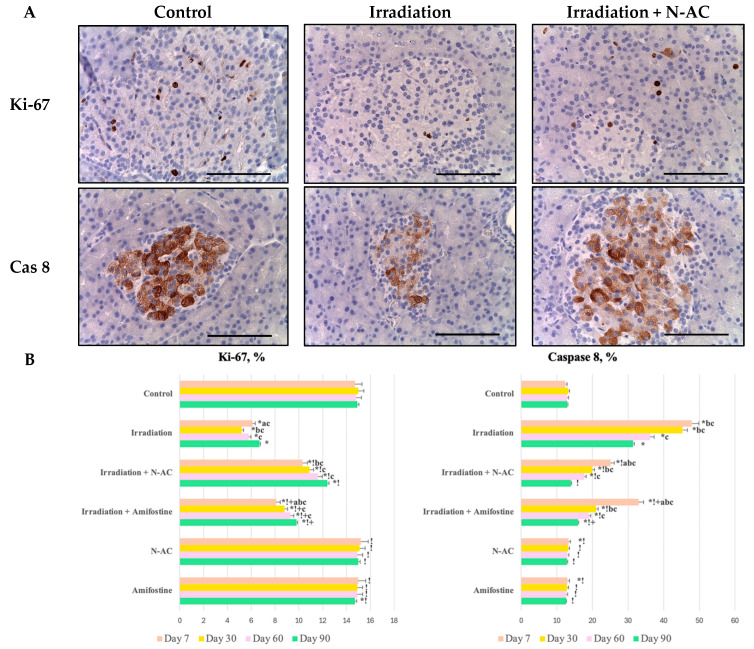
Pancreas of the control and experimental groups. Immunohistochemical reactions with antibodies to Ki-67 and caspase-8 (Cas 8), magn. ×400 (**A**). Scale bar—25 μm. Distribution of Ki-67 and caspase-8 expression in the pancreas at days 7, 30, 60, and 90 (**B**). Data are presented as mean values (range), with Kruskal–Wallis test and Mann–Whitney U-test values provided. SOD—superoxide dismutase, MDA—malondialdehyde, N-AC—N-Acetylcysteine. Statistically significant differences (*p* < 0.05): a—day 7 vs. day 30 within the same group, b—compared to day 60 within the same group, c—compared to day 90 within the same group; *—compared to control; !—compared to the irradiation group at the same time point; +—Amifostine vs. N-AC.

An immunopositive reaction with antibodies against Ki-67 was detected in the nuclei of the exocrine pancreatic cells and endocrine cells in the pancreatic tissue samples from the control group. One week after the fractional local electron irradiation of the pancreas, a sharp decrease in Ki-67-positive exocrine pancreatic cells and endocrine cells was observed compared to the control values. In the group that received N-AC before irradiation, moderate immunolabeling for Ki-67 was noted in the endocrine and exocrine cells of the pancreas (Figure 4).

Moreover, a weak positive staining of exocrine pancreatic cells and endocrine cells for caspase-8 was observed in the pancreatic tissue samples from the control group, appearing as dark brown granules in the cellular cytoplasm. Pronounced immunolabeling of the cytoplasm with antibodies against caspase-8 was noted one week after fractional local electron irradiation.

The exocrine pancreatic cells and endocrine cells in the samples from group III exhibited a moderate immunopositive reaction with antibodies against caspase-8 (Figure 4).

In the early stages, N-Acetylcysteine was more effective than Amifostine in some parameters, whereas in the irradiation group, no positive trends in the studied parameters were observed throughout the experiment.

**Figure 5:**
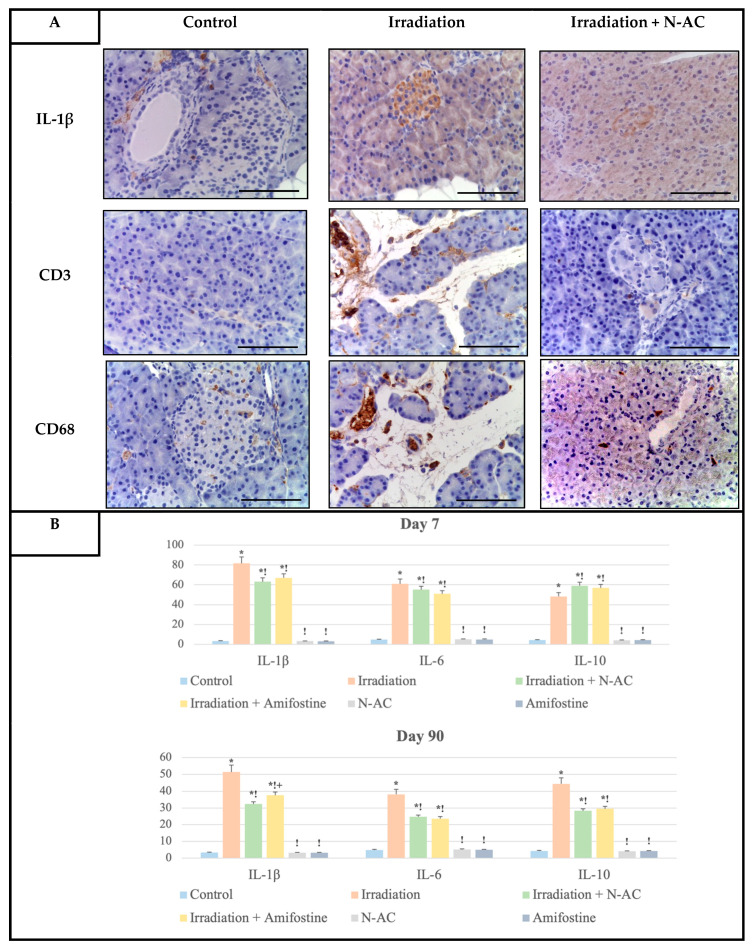
Immunohistochemical assessment of inflammation markers IL-1β (pro-inflammatory cytokine), CD3 (T-lymphocytes), and CD68 (macrophages) in the pancreas of the control and experimental groups, magn. ×400 (**A**). Scale bar—25 µm. Distribution of IL-1β, IL-6, and IL-10 expression levels in the pancreas of the control and experimental groups on days 7 and 90 (**B**). y-axis—percentage of positive cells in one islet in relation to the total number of endocrine cells. Data are presented as mean values (ranges), with Kruskal–Wallis test and Mann–Whitney U-test values provided. SOD—superoxide dismutase, MDA—malondialdehyde, N-AC—N-Acetylcysteine. Statistically significant differences (*p* < 0.05): *—compared to control; !—compared to the irradiation group at the same time point; +—Amifostine vs. N-AC.

An intense membrane staining was observed, predominantly in the endocrine cells of the pancreatic islets, after electron irradiation in the IHC study with antibodies against pro-inflammatory (IL-1β, IL-6) and anti-inflammatory (IL-10) cytokines (Figure 5).

The number of positively stained cells varied over time, with the highest proportion observed in the first week, followed by a decline by the third month. At the same time, weak immunohistochemical staining was noted at all time points in some cells of the exocrine part of the pancreas. Based on cytological characteristics, IL-1β and IL-6 immunolabeling were detected in large cells, likely of monocytic origin, as well as in mast cells, which were primarily located perivascularly (Figure 5).

In the group that received N-AC before local electron irradiation, there was an expression of both pro-inflammatory (IL-1β, IL-6) and anti-inflammatory (IL-10) cytokines, which was detected at all time points in some endocrine cells of the pancreatic islets and a few acinar cells (Figure 5).

In immunohistochemical reactions with antibodies against CD3+ T-lymphocytes, single CD3-positive cells were detected one week after electron irradiation, similar to the control group (1 point). However, their number had increased (2 points) by the third month. Interestingly, the immunolabeling of these cells was mainly observed in expanded interlobular septa along blood vessels, as well as in a few endocrine cells, where a dispersed membrane staining pattern was noted in some pancreatic islets (Figure 5).

When analyzing the results of the immunohistochemical reactions with antibodies against CD68, single CD68+ macrophages were observed in the control group during the first week of the experiment (1 point). Local electron irradiation led to a gradual increase in their number throughout the experiment, reaching approximately six cells per field of view by the third month (2 points). Notably, these cells were predominantly located perivascularly (Figure 5).

## 4. Discussion

Exposure to ionizing radiation leads to the development of signs of acute or subacute radiation-induced pancreatitis [14]. This occurs because of the activation of both direct and indirect mechanisms of radiation damage. The direct pathway is associated with electron beam-induced damage to nuclear and mitochondrial DNA, resulting in single- and double-strand breaks or cross-links [15]. Single-strand breaks are conditionally repairable and can be restored using the template of the second intact strand. However, their accumulation triggers the activation of the MAPK/Jnk/p38 complex involving transforming growth factor-α (TGF-α) and tumor necrosis factor-α (TNF-α), leading to the “bystander effect” in surrounding normal tissues and the induction of cell apoptosis [16].

At the same time, the indirect effects of radiation on the pancreas are driven by the generation of free radicals, including reactive oxygen species (hydrogen peroxide H_2_O_2_, singlet oxygen ^1^O_2_, superoxide anion radical O_2_•^−^, and hydroxyl radical OH•), reactive nitrogen species, and lipid peroxidation products (LOOH), among others [17]. This leads to the development of oxidative stress, resulting in further damage to intracellular organelles and macromolecules. These changes were confirmed by our assessment of the endogenous redox system: we observed an increase in MDA levels and a decrease in SOD activity after electron irradiation, which is consistent with the findings of other researchers [18].

According to our data, oxidative damage to pancreatic cells in response to electron irradiation led to structural disruption of the pancreas and the death of islet cells. In the morphological study, we identified signs of acute or subacute radiation-induced pancreatitis [19]. The observed changes, including cell vacuolization, focal necrosis, inflammation, edema, and hemorrhages, were accompanied by a reduction in morphometric parameters (number, diameter, and area of islets) and a decrease in the number of endocrine cells. Additionally, we observed an increase in the number of cells positively stained for caspase-8, along with a decrease in their proliferation (↓Ki-67).

The observed changes indicate the death of cells, primarily in the endocrine part of the pancreas, which, because of its high proliferative activity, exhibits greater radiosensitivity compared to exocrine cells and stromal components, in accordance with the Bergonié–Tribondeau law [20]. Elevated caspase-8 levels suggest the activation of the extrinsic apoptotic cascade, leading to the apoptotic death of α-cells and β-cells, as identified by IHC staining with insulin and glucagon antibodies. This finding is consistent with previous research, as cell death occurs through the binding of the CD95/APO-1 complex to the Fas ligand, followed by caspase-8 activation and subsequent apoptosis [21].

An interesting aspect is the study of the inflammatory response to local electron irradiation. DNA damage, combined with the death of acinar and endocrine cells, leads to the accumulation of damage-associated molecular patterns (DAMPs) in the pancreas, which interact with macrophage-like cells and induce TNF-α gene expression. The accumulation of this pro-inflammatory cytokine in the pancreas triggers the recruitment of leukocytes, primarily new macrophages, neutrophils, and mast cells [22]. This explains the high levels of CD68-positive cells in the pancreatic stroma after electron irradiation, as observed in our study. Subsequently, the recruited immune cells express a large number of new pro-inflammatory cytokines, including TGF-β1, IL-1β, IL-6, VEGF-A, PDGF, IL-33, and NF-κB, among others [9].

In response to the sharp increase in IL-1β and IL-6 levels, identified in our study, inflammation is initiated, consisting of two components: the capillary–parenchymal block (vascular reaction) and cellular inflammatory infiltration. At the same time, the expression of the anti-inflammatory cytokine IL-10 was reduced, which may indicate an imbalance between pro- and anti-inflammatory factors in the pancreas in response to electron irradiation. As a result, acute and/or subacute inflammation of the pancreas develops, with specific features of electron-induced damage being described for the first time in this study.

Significant expression of genes responsible for the synthesis of pro-inflammatory cytokines leads to the activation of four major signaling pathways, whose modulation enhances inflammation, cytokine synthesis, and immune cell migration. The most well-studied pathway in response to radiation exposure is the MAPK pathway. Activation of the MAPK and Jnk/p38 complex in response to extracellular stress signals modulates cell proliferation, survival, apoptosis, and cytokine synthesis. Subsequently, MAP/ERK kinase-1 (MEK-1) may contribute to the chronicity of pancreatitis and the development of fibrosis, likely through the action of angiotensin-2 [23].

In the acute phase of radiation-induced pancreatitis, activation of the JAK/STAT signaling pathway occurs in response to increased IL-6 levels. This pathway regulates cell proliferation, differentiation, and inflammation by inducing the synthesis of TNF-α, IL-17, IL-1β, and FGF-2 [22]. Additionally, activation of PI3K along with downstream AKT and mTOR occurs, which regulates the cell life cycle and may potentially enhance trypsinogen activation in acinar cells, suggesting the possibility of autolysis under radiation exposure [24].

One of the key molecular mechanisms of inflammation and fibrosis in the pancreas during pancreatitis is the TGF-β1/SMAD pathway [25]. This pathway likely induces the inflammatory response through dysregulation of the micro-RNA-217-SIRT1 pathway and is also capable of activating the previously mentioned MAPK and PI3K pathways, thereby amplifying inflammation [26]. Additionally, TGF-β1/SMAD-2 and -3 and ERK may contribute to fibrosis in the long term [22].

In this study, we also evaluated the effectiveness of N-Acetylcysteine (N-AC) as a radioprotector. We observed a reduction in the severity of the inflammatory response compared to irradiated animals, protection of the proliferative–apoptotic balance in the pancreas, and preservation of organ structure. These properties of N-AC are likely related to its antioxidant effect, which involves both the scavenging of hydroxyl radicals and the stimulation of endogenous antioxidant defense enzymes, as confirmed by the measurement of SOD and MDA levels. These findings are consistent with other studies [27]. It is interesting that after N-AC administration without irradiation, a decrease in the MDA level in the pancreas was noted below the control, which may be associated with the inhibition of aging processes that are accompanied by the oxidation of lipid molecules and the formation of MDA in the healthy pancreas [28].

These mechanisms are attributed to the presence of a Thiol group in the structure of N-AC, which is capable of directly binding reactive oxygen species, such as OH, H_2_O_2_, HOCl, and others [18,29]. As a result, the number of free radicals is significantly reduced, leading to the effects of N-AC, including anti-inflammatory, anti-apoptotic, and cytoprotective actions [13]. Moreover, based on the assessment of delayed effects, an indirect reparative property of this compound was identified, as it accelerates pancreatic recovery (particularly in the islets) after electron exposure. This effect can be considered indirect, as it is also mediated by the reduction in free radicals at the early stages.

It should be noted that Amifostine (Ethiofos), a drug with proven efficacy in radiation-induced damage, is widely recommended for use in clinical oncology practice [11]. However, its cost, route of administration, and toxicity limit its application. In contrast, N-AC appears to be not only a more convenient and simpler alternative but also demonstrates greater efficacy compared to Amifostine. This makes N-AC a potentially more favorable choice as a radioprotector during electron irradiation, with its effectiveness confirmed in this study by nearly every research method employed.

Thus, for the first time, we identified that electron irradiation leads to the activation of the aforementioned molecular cascades, resulting in a sharp increase in cytokine expression and inducing cellular inflammatory infiltration by macrophages (CD68+) and mast cells. This also highlights the crucial role of the microenvironment in the development and maintenance of the inflammatory response. Additionally, we observed a significant increase in T-lymphocytes, as demonstrated by IHC analysis with antibodies against their general marker CD3. These changes were effectively prevented by the pre-irradiation N-AC administration. The action of this compound is associated with a reduction in free radical levels and the stimulation of the redox system, which limits the apoptotic death of both exocrine and endocrine pancreatic cells and prevents the development of radiation-induced pancreatitis. Furthermore, our data suggest that N-AC offers multiple advantages over conventional radioprotective agents.

## 5. Materials and Methods of This Research

### 5.1. Animals and Design

In the experimental study, male Wistar rats, aged 8–9 weeks (*n* = 180), were used. The animals were housed under a 12 h light/dark cycle, with air conditioning at 23 °C and 40–60% humidity. The rats were provided with a standard diet and access to water ad libitum. The rats were kept in plastic cages lined with a layer of absorbent material (rice husk), which served as nesting material. During the long-term experiment, the animals were housed in pairs per cage to eliminate the potential effects of prolonged solitary confinement on behavior.

The Wistar rats (Rattus Wistar; *n* = 180) were divided into six experimental groups:

I—control (*n* = 20) rats were injected with 0,9% NaCl solution;

II—irradiation (*n* = 40) rats were subjected to fractional local irradiation with electrons at a total irradiation dose of 30 Gy;

III—irradiation + N-AC (*n* = 40) rats were administered N-Acetylcysteine intraperitoneally at a dose of 120 mg/kg 1 h before local electron irradiation, with a total irradiation dose of 30 Gy;

IV—irradiation + AMI (*n* = 40) rats were administered Amifostine intraperitoneally at a dose of 150 mg/kg half an hour before local electron irradiation, with a total irradiation dose of 30 Gy;

V—N-AC (*n* = 20) rats were administered N-Acetylcysteine intraperitoneally at a dose of 120 mg/kg;

VI—AMI (*n* = 20), rats were administered Amifostine intraperitoneally at a dose of 150 mg/kg.

Five animals from groups I, V, and VI and ten animals from groups II, III, and IV were euthanized by administering high doses of anesthetic (Ketamine + Xylazine) four times: 1 week, 1 month, 2 months, and 3 months after the start of the experiment. The start date of the experiment for the irradiated groups was considered the day of the last fraction. For non-irradiated animals, the start date of the experiment was the same.

### 5.2. Irradiation Model

The animals underwent partial local electron irradiation in the dorsal region at the projection area of the pancreas. The irradiation parameters were as follows: total irradiation dose of 30 Gy in 5 Gy fractions with a one-day break between each one, dose rate of 1 Gy/min, energy of 10 MeV, and frequency of 9 Hz, with a field size of Ø 50 mm. The procedure was performed using a “NOVAC-11” linear accelerator from the Radiology Department of the Experimental Sector of the A.F. Tsyb Medical Radiological Research Center (MRRC), Obninsk, Russia.

### 5.3. Drugs

The animals in groups III and V were administered a 10% water solution of N-Acetylcysteine (100 mg/mL, Zambon, Milano, Italy), at a dose of 120 mg/kg, in the form of intraperitoneal injections. The scientific literature uses both standard doses of the drug (100–500 mg/kg of rat body weight) and doses significantly higher than recommended (600–1000 mg/kg). In our pilot study, we found that a dose of 120 mg/kg was sufficient to produce an antioxidant effect while being better tolerated than higher concentrations of the drug. For radioprotection, N-acetylcysteine (N-AC) was administered to the laboratory animals 60 min (the time required for absorption and reaching the maximum concentration of the drug in the blood) before each fraction of local electron irradiation. The animals in groups IV and VI were administered Amifostine (WR2721, 100 mg of white powder for i/v injections, MedChemExpress LLC., Shanghai, China) intraperitoneally at a dose of 150 mg/kg in the form of a powder dissolved in phosphate-buffered saline according to the standard protocol [30]. The drug doses were therapeutic according to the instructions and were calculated for rats using interspecies dose conversion coefficients for preclinical studies.

### 5.4. Biochemical Blood Assay

Blood glucose levels were measured by cannulating the aorta with a polyethylene catheter using the One Touch Verio Reflect glucometer (LifeScan Europe GmbH., ZUG, Switzerland). Insulin and amylase levels were determined using the PRIETEST TOUCH biochemical analyzer (DIXION, Moscow, Russia).

### 5.5. Oxidant-Antioxidant Assay

Pancreatic homogenate was obtained by homogenizing 1 g of tissue in 4,5 mL of cold potassium buffer (pH = 7.4). The solution was then centrifuged at 13,000 rpm for 10 min at 4 °C. The supernatant was subsequently stored at −80 °C. The levels of malondialdehyde (MDA) as a biomarker of lipid peroxidation and superoxide dismutase (SOD) in the pancreatic homogenate were assessed using ELISA Kit assays (LifeSpan BioSciences, Newark, CA, USA).

### 5.6. Morphological Block

#### 5.6.1. Histologic Research

Pancreatic tissue fragments were fixed in buffered formalin solution, processed automatically, embedded in paraffin blocks, and sectioned into serial slices with a thickness of 2 μm. The sections were then deparaffinized, dehydrated, and stained with hematoxylin and eosin. The histological specimens were examined under a Leica DM2000 microscope with microphotography (Leica Microsystems, Wetzlar, Germany). The evaluation of the specimens was conducted according to standard histological criteria [19].

#### 5.6.2. Morphometric Analysis

Morphometric analysis was performed in 10 randomly selected microscopic fields at ×400 magnification on 5 random sections from each sample using a Leica Application Suite image analyzer (Leica Microsystems, Wetzlar, Germany) v. 4.9.0 and ImageJ software v. 1.54p (Bethesda, MA, USA). The following parameters were calculated: the average area and diameter of pancreatic islets, their number per 1 mm^2^, and the percentage of endocrine cells within a single islet [31].

#### 5.6.3. Immunohistochemical (IHC) Assay

For IHC analysis, the following primary antibodies were used: monoclonal antibodies against Ki-67 (Clone MM1, ThermoFisher, Waltham, MA, USA) and caspase-8 (Clone B.925.8, ThermoFisher, Waltham, MA, USA) and polyclonal antibodies against the cytoplasmic antigen of β-cells–insulin (EP125, 1:300, Cell Marque, Rocklin, CA, USA), the cytoplasmic antigen of α-cells–glucagon (259A-15, 1:100, Cell Marque, Rocklin, CA, USA), Interleukin-1β (IL-1β; 1:100, ThermoFisher, Waltham, MA, USA), Interleukin-6 (IL-6; 1:100, ThermoFisher, Waltham, MA, USA), and Interleukin-10 (IL-10; 1:100, ThermoFisher, Waltham, Massachusetts, USA). Ready-to-use (RTU) polyclonal antibodies (Leica, Wetzlar, Germany) were applied for CD3 (T-lymphocytes) and CD68 (macrophages). For secondary antibody detection, a universal two-component HiDef Detection™ HRP Polymer system (Cell Marque, Rocklin, CA, USA) was used, including anti-IgG mouse/rabbit antibodies, horseradish peroxidase (HRP), and DAB substrate. Cell nuclei were counterstained with Mayer’s hematoxylin.

The quantitative data obtained from the IHC assessment of insulin- and glucagon-positive cells were converted into scores according to the following scale:

0 points—less than 5% of positive cells per one pancreatic islet;

1 point—6–25% of positive cells per one pancreatic islet;

2 points—26–50% of positive cells per one pancreatic islet;

3 points—more than 50% of positive cells per one pancreatic islet [19].

The counting of Ki-67-, caspase-8-, and IL-immunopositive cells was performed in 10 randomly selected fields of view at ×400 magnification (expressed as a percentage). Thus, the number of immunopositive cells in the islet was calculated, which was divided by the total number of cells in one islet and multiplied by 100%. The number of CD+ cells was determined by computer morphometry in 10 fields of view, and the obtained morphometric data were converted into scores on a scale from 1 to 3 as follows:

1 point—fewer than 3 CD+ cells;

2 points—from 4 to 6 CD+ cells;

3 points—from 7 to 10 CD+ cells per field of view under a light microscope.

### 5.7. Statistical Significance

The data obtained from the counts were processed using SPSS 12 for Windows software (IBM Analytics, Armonk, NY, USA). The results are expressed as mean ± standard deviation (SD). The Shapiro–Wilk test was used to assess the normality of distribution. For comparisons between the study groups with a distribution deviating from normal, the Kruskal–Wallis test with Dunn’s post hoc test was applied. Multiple comparisons were performed using the Mann–Whitney U-test. A *p*-value of ≤0.05 was considered statistically significant.

## 6. Conclusions

For the first time, a comprehensive analysis of biochemical, morphological, and immunohistochemical markers in the pancreas of a large cohort of electron-irradiated animals has been conducted, including both acute and delayed effects of electron exposure. The crucial role of interleukins in shaping both the cellular and vascular components of the inflammatory response has been identified. Additionally, the radioprotective properties of N-Acetylcysteine during electron irradiation of the pancreas have been evaluated for the first time, and its effectiveness in reducing both acute and late complications of electron therapy has been demonstrated. Thus, it can be concluded that N-Acetylcysteine is capable of effectively suppressing the inflammatory response in the pancreas.

## Data Availability

This study did not generate publicly available archival data.
